# Nano-Hydroxyapatite Isolation and Characterisation of *Echinometra mathaei* from the Persian Gulf

**DOI:** 10.21315/tlsr2023.34.2.12

**Published:** 2023-07-21

**Authors:** Ali Rajabiyan, Luigi Vaccaro, Amanollah Zarei Ahmady

**Affiliations:** 1 Marine Pharmaceutical Science Research Centre, Ahvaz Jundishapur University of Medical Sciences, Ahvaz, Iran; 2 Laboratory of Green S.O.C. – Dipartimento di Chimica, biologia e Biotecnologie, Università degli Studi di Perugia, Via Elce di Sotto 8, 06123, Perugia, Italy; 3 Nanotechnology Research Centre, Ahvaz Jundishapur University of Medical Sciences, Ahvaz, Iran; 4 Department of Medicinal Chemistry, Faculty of Pharmacy, Ahvaz Jundishapur University of Medical Sciences, Ahvaz, Iran

**Keywords:** Natural Sources, Nano-Hydroxyapatite, Sea Urchin Shell, *Echinometra mathaei*, Hydrothermal

## Abstract

The study focuses on the preparation and characterisation (physicochemical and mechanical) of hydroxyapatite [Ca_10_(PO_4_)_6_(OH)_2_] (HA) from sea urchin, *Echinometra mathaei*. Therefore, nano-sized HA prepared from sea urchin shells were collected from beaches of the Persian Gulf in Iran. Sea urchin shells were found a source of calcium carbonate in the form of aragonite (calcite) that crystallised in an organic matrix. HA is one of the polymers used in coating the nanoparticles extracted from various sources. The calcined aragonite converted to nanosized hydroxyapatite powder by chemical reaction with orthophosphoric acid while maintaining stoichiometry, Ca/P = 1.667 at 80°C. To determine the purity of the nano-hydroxyapatite (nHA) numerous analytical procedures were used. Fourier transforms infrared spectroscopy (FTIR) confirmed the presence of the peak of 961 cm^−1^ is related to the symmetric tensile band of the P-O bond, and the peak of 1038 cm^−1^ and 1091 cm^−1^ is related to the tensile solid absorption of the PO_4_ as functional groups of nHA. The nanocrystalline HA can be observed from the SEM images. Thermogravimetric analysis (TGA-DTA) demonstrates the thermal stability of nHA powder. The results show successful isolation and characterisation study of this crucial nano-material shows it is valuable in biomedical applications, particularly in bone tissue engineering. Indeed, its fabrication is easy and economical.

**Highlights**

- Sea urchin shells are composed mainly of calcium carbonate and can be used as the calcium precursor source for hydroxyapatite (HA).
- The nano-hydroxyapatite (nHA) powder generated is bulky in nature, since it is formed up of nanocrystalline molecules that form a microcrystalline molecule.
- The nHA powder average particle size (length) was in the range of 5 nm to 1 μm.

## INTRODUCTION

Several bioactive chemicals and minerals, including calcium carbonate, chitin, colours and proteins, are abundant in marine shell debris. Due to off-odour and mineral concentration in landfills, this waste material is now underutilised and contributes to serious environmental problems (Hou *et al*. 2016). In addition, marine species such as eggshells, sea urchins, sponges, coral, nacre, mussels and land snails can used as natural resources in medicine, pharmaceutical manufacture and biological chemicals (Şahin *et al*. 2015; Cahyaningrum *et al*. 2018; Nabavi *et al*. 2017).

Sea urchins, sand dollars, starfish and sea cucumbers are all members of the Phylum Echinodermata. Urchins can be found in a wide range of marine settings, from polar to tropical temperatures, and at depths of up to 5,000 m. Because it shields itself against degradation, most of its spines are long and sharp, and Spines with a diameter of up to 1 cm and a length of up to 30 cm (Naleway *et al*. 2016). Around 1,000 different species of marine urchin have identified around the world (Rahim & Nurhasan 2016). Most species remain in the relatively small tropical and temperate waters (Ettensohn 2017).

The most crucial inorganic biomaterial in biomedical applications is HA (Khandelwal & Prakash 2016). HA has high biocompatibility and the property of being osteoconductive, which means it can induce bone development. Ca_10_(PO_4_)_6_(OH)_2_ is the chemical formula for HA, with a molar Ca/P ratio of 1.67 (Cahyaningrum *et al*. 2018). Pores, bioactivity, biocompatibility and osteoconductive properties are all present in HA. Cao, Ca(NO_3_)_2_, Ca(OH)_2_, CaCO_3_ and CaCl_2_ are familiar sources of synthetic calcium utilised to manufacture HA. HA is the principal inorganic constituent of human bones and teeth; It is readily found in nature, easily available, at an affordable price, and has a low environmental effect when it comes to restoring and remediating natural resources (Venkatesan & Kim 2014).

Precipitation, radiofrequency thermal plasma, reverse microemulsion, an emulsion liquid membrane system, the sol-gel method, microwave processing, hydrothermal procedures, wet chemical precipitation and calcium phosphate hydrolysis are some of the synthetic processes utilised to make nHA (Komalakrishna *et al*. 2017). The traditional precipitation process used to make synthetic HA types (Ağaoğullari *et al*. 2012). Synthetic nHA manufacture, on the other hand, frequently necessitates the employment of toxic chemicals, aging procedures and an unbalanced stoichiometric ratio (Venkatesan *et al*. 2015). It could be due to the several precursors required for syntheses, severe agglomeration, costly equipment and phase impurities in nHA (Elmi *et al*. 2018).

Although there is not any study in the Persian Gulf in this regard, some studies there are in another regions. Gómez Vázquez *et al*. (2020) reported the synthesis of angular distance from echinoderm spines was collected at a landfill in Baja California. This indicates that this research may be a starting point for the creation of HA on an industrial scale, by rising synthesis parameters. whereas this research proves that there are higher parameters to be found by victimisation in completely different temperatures, there’s still progress to be created in the production of HA while using lower biogenesis energy.

Alvarez-Lloret *et al*. (2010) reported analysing the crystallographic relationships during the hydrothermal conversion of a calcitic echinoderm spine into a mineral. We identified a pseudomorphic mineral replacement mechanism involving a superficial dissolution of calcite and a subsequent overgrowth of oriented carbonated HA nanocrystals. Cross-section images of these converted spines show that the dimensions of the HA crystals increase the further they are from the outer surface.

Mancilla Sanchez *et al*. (2019) reported a simple, quick and viable way to produce HA from waste by-products of the seafood industry. This work shows that we can take advantage of raw materials derived from processing seafood by giving it added value for the biomedical business. In the other study, Shavandi *et al*. (2016) reported converting waste kina shells to hydroxyapatite using optimum process conditions while protecting its natural porous structure. Results indicated that a temperature higher than 650°C is sufficient to remove organic matter without damaging the structure (Shavandi *et al*. 2016).

Due to low-cost chemical synthesis, HA generated from marine organisms is more cost-effective than commercially available HA. Natural sources have the advantage of inheriting some raw material qualities (Komalakrishna *et al*. 2017). The most chemical approach in an aqueous solution is the most often utilised method of nHA production (Venkatesan & Kim 2014). Synthetic HA derived from reagent chemicals, on the other hand, is another alternative source, but it lacks crucial trace elements (such as strontium) (Şahin *et al*. 2015).

HA can be used in dentistry, bone implantation, tissue engineering, orthopedic composites and pharmaceutical delivery systems (Szatkowski *et al*. 2015; Mohd Pu’ad *et al*. 2019). Because it resembles rough bodily tissues, HA has remarkable biocompatibility with bone cells and tissues. Calcium biomaterials, such as powders, granules, dense and porous blocks, and other composites, are frequently employed in medicinal applications *(*Ozyegin *et al*. 2012). Its chemical composition and crystallographic structure are comparable to those of the biomineral in natural tissue; HA generated artificially can used to heal injured bone or teeth (Komalakrishna *et al*. 2017).

**Figure 1:**
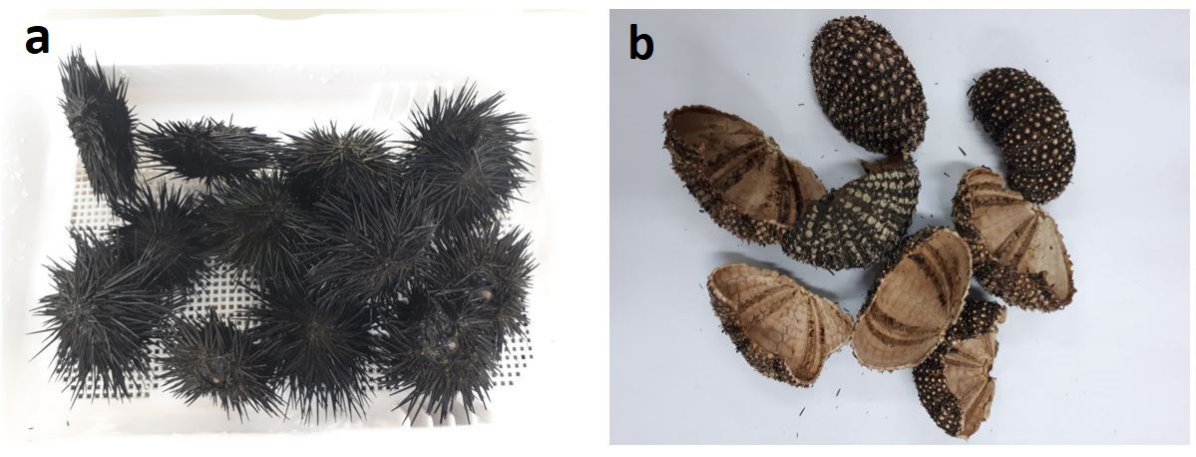
Optical image of (a) live sea urchins, and (b) dead sea urchins.

Sea urchin (*E. mathaei*) shells are used as HA source in this survey. *E. mathaei* is a common type of sea urchin found in the Persian Gulf (Fig. 1) and it may be found in vast quantities throughout the coastline. The Persian Gulf is found among 24° to 30° 30′ N latitude and 48° to 56° 25′ E longitude. The current Persian Gulf could be a little a part of an enormous water house known as the Persian ocean within the remote past because it embraced the whole Makran (Oman) ocean and a region of the ocean up to the Indus River. The Persian Gulf is the warmest water expanse best known with a dry semi-tropical climate. Its widest part reaches 180 miles and its depth is from 10 m–30 m in the west to 93 m which is its most profound point 15 km far from the Island, big Tonb. The low depth of the gulf constantly causes the land to advance into the sea (Persian Gulf Study Centre n.d.). This research aimed to prepare nano-hydroxyapatite from the Persian Gulf sea urchin and its characterisation study.

## MATERIALS AND METHODS

### Materials and Reagents

Orthophosphoric acid (H_3_PO_4_) purchased from EMSURE^®^ Merck, Germany. All other compounds were analytical grade that utilised without additional purification and bought from Sigma-Aldrich and Merck. Sea urchin shells are collected from Souza port city the Persian Gulf. Fourier-transform infrared (FTIR) spectrophotometer (Perkin Elmer, Waltham, MA, USA), scanning electron microscope (HITACHI S2600N-type SEM, Japan) and Thermogravimetric apparatus (Perkin Elmer Elan DRC 6000; TG-DTA, USA) were used for analysis.

### Sample Preparation

**Figure 2:**
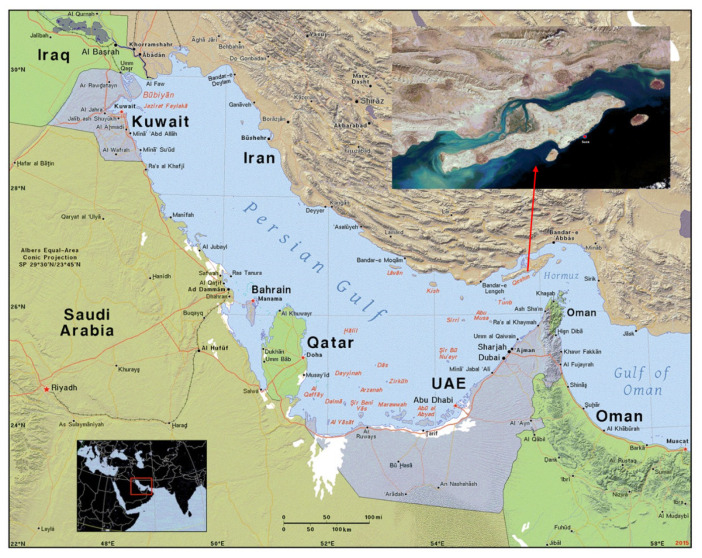
Location map of the study area (IRAN). *Source:* https://ian.macky.net/pat/map/pers/pers.html

In June 2018, sea urchins of the *E. mathaei* species obtained physically at a depth of 10 m near the Souza port city the Persian Gulf (26°46′51″ N and 56°03′47″ E) (Fig. 2). To establish the genus and species of sea urchins, the appearance characteristics of the sea urchins has been analysed, and Price (1983) employed a regional identification key to establishing the sex and species type used. The samples are brought to the laboratory frozen. The samples were dissected first, and their internal organs were separated. The shells then desalted by being rinsed twice in cold water. They were placed in a freeze-dryer for 24 h at −40°C to dry and remove moisture.

### Synthesis of CaO from Sea Urchin Shell

CaCO_3_ is the most abundant element in the sea urchin shell, accounting for almost 94% of the total weight. Sea urchin shells employed to synthesise CaO in this approach. Ten sea urchins were used in this research. To remove any filth from the shells, they washed and cleaned the shells with tap water. The shells were crushed into tiny pieces before being ground by hand in a mortar. A 75 μm sieve used to filter ground powders. The extraction procedure used was consistent with the Komalakrishna *et al*.’s (2017) study.

Beyond 900°C, the carbon dioxide in the sea urchin shell transformed into calcium oxide. The expected reaction is:


(1) $${\text{CaCO}}_{3}\to \text{CaO}+{\text{CO}}_{2}↑$$

### Synthesis of nHA from Sea Urchin Shells

In a beaker, a defined amount of calcined sea urchin shell powder dissolved dated in distilled water and stirred at 80°C with a magnetic stirrer. The amount of calcium in the calcined sea urchin shell used to determine the stoichiometry. CaO is transformed into Ca(OH)_2_ in this reaction, as shown in Equation 2:


(2) $$\text{CaO}+{\text{H}}_{2}\text{O}\to \text{Ca}{\left(\text{OH}\right)}_{2}+\text{Heat}$$

The orthophosphoric acid (H_3_PO_4_) reagent grade was added to the Ca(OH)_2_ solution. Drop-by-drop, the solution was supplied precisely to maintain a stoichiometric Ca/P molar ratio of 1.667 and lower the pH to 8.5. It had agitated for 2 h at 200 rpm, which aids in precipitation production. At this point, the production of precipitation were noticed. Furthermore, the final product dehumidified overnight in a hot air oven at 100°C. This process outcome is predicted as follows:


(3) $$10\text{Ca}{\left(\text{OH}\right)}_{2}+6{\text{H}}_{3}{\text{PO}}_{4}\to {\text{Ca}}_{10}{\left({\text{PO}}_{4}\right)}_{6}{\left(\text{OH}\right)}_{2}+18{\text{H}}_{2}\text{O}$$

The precipitate filtered with a membrane filter, then rinsed with double deionised water before being filtered again. The precipitate dried for another 2 h at 100°C in the oven. The dried precipitate calcined for 2 h at 1100°C in a tube furnace. At the end of the process, the crystalline powder agglomerates were discovered in the furnace.

### Characterisation of Nano-Hydroxyapatite (nHA)

The scanning electron microscope (SEM) was used to characterise the morphology of the nHA powder, operating at 25 kV in a vacuum. Before the analysis, the samples were fixed on a copper specimen holder with carbon tape and protected with a gold thin film to make them conductive before testing the scanning electron microscope with field emission (Quanta 200; FE-SEM). Thermogravimetric apparatus results used to determine the HA’s thermostability and losing weight. In the presence of air, a heating rate of 10°C/min used to reach a temperature of 1000°C. FTIR spectrophotometer used to investigate the functional groups presenting in the sea urchin shell. Two milligrams (2 mg) of each sample mixed with 98 mg of KBr (Potassium bromide) powder. The infrared spectra measured in medium infrared (MIR) between 400 cm^−1^–4000 cm^−1^ with a 4 cm^−1^.

## RESULTS

### Fourier-transform infrared (FTIR) Analysis

**Figure 3:**
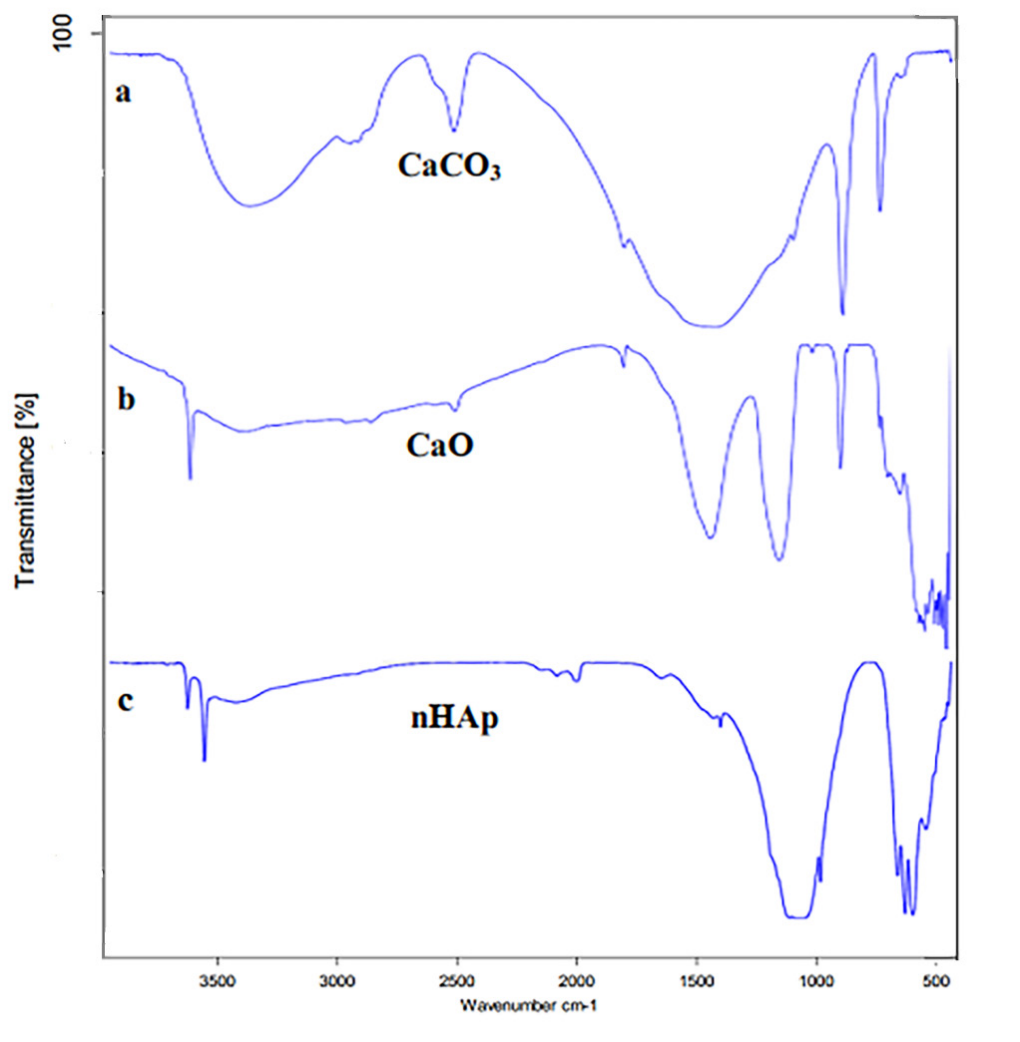
FTIR spectra of (a) CaCO_3_; (b) CaO and (c) nHA.

Fig. 3 represents the FTIR spectra of the hydroxyapatite samples.

Pure calcium carbonate made from sea urchin shells as shown in the FTIR spectrum, tensile solid adsorption of about 1417 cm^−1^ (C-O bond), and a relatively flexural solid adsorption of about 875 cm^−1^ (C-O bond) confirms the formation of calcium carbonate. In the FTIR spectrum of calcium oxide, the existence of a peak of 427 cm^−1^ related to Ca-O bond, a peak of 876 cm^−1^ and 1430 cm^−1^ related to C-O and a peak of 3642 cm^−1^ related to O-H bond, and the removal of the carbonyl peak from the prepared calcium carbonate spectrum indicate the decarbonisation of calcium carbonate to form calcium oxide.

In the FTIR spectrum of nHA, the peak of 961 cm^−1^ is related to the symmetric tensile band of the P-O bond, and the peak of 1038 cm^−1^ and 1091 cm^−1^ is related to the tensile solid absorption of the PO_4_ functional group. The adsorbents appearing in the regions of 3570 cm^−1^ and 3641 cm^−1^ show the O-H tensile vibrations of nHA and water in its system, respectively. Also, the presence of 1384 cm^−1^ and 1637 cm^−1^ bands are peaks related to (C-O bond) of the carbonate functional group, and this suggests that carbonate ions have taken the position of phosphate ions in the apatite structure.

### SEM Micrograph

**Figure 4:**
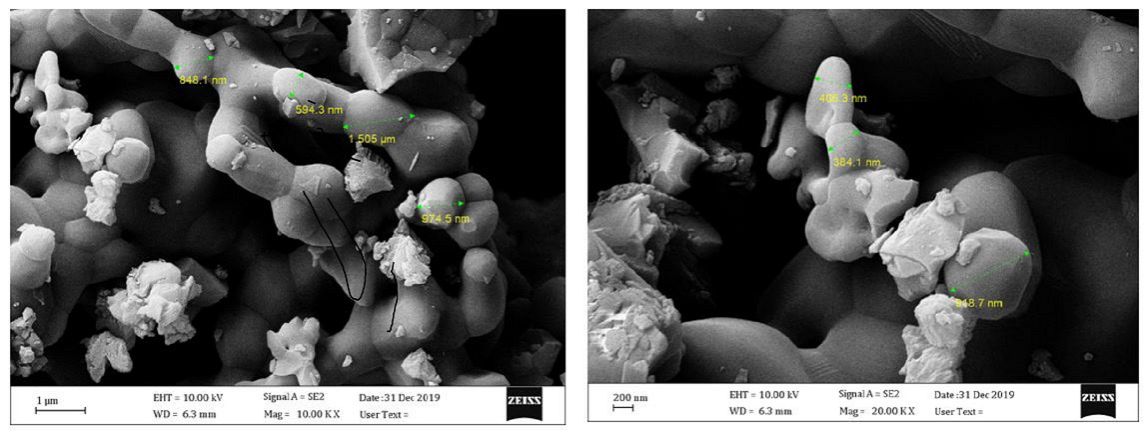
SEM images of nHA.

Fig. 4 shows scanning electron microscope pictures of synthesised nHA powder captured at 200 nm and 1 μm magnification, showing nanocrystalline nHA. The nHA powder generated is bulky in nature, since it is formed up of nanocrystalline molecules that form a microcrystalline molecule. SEM micrographs revealed that the average particle size (length) was in the range of 5 nm to 1 μm.

### TG–DTA Analysis

**Figure 5:**
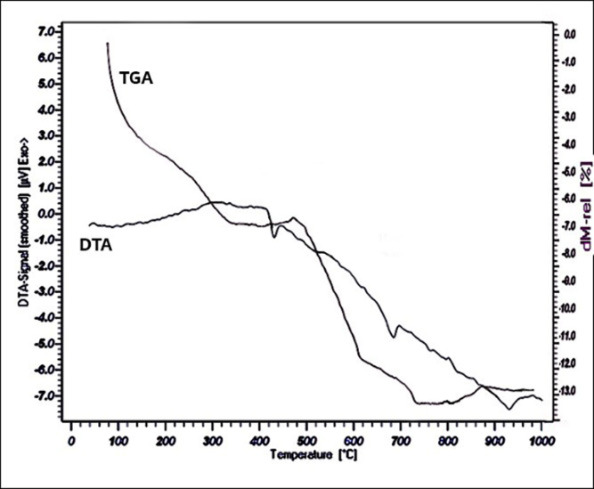
Differential and gravimetric thermal analysis (DTA/TGA) of the nHA.

Thermal experiments used to analyse the chemical changes in the nHA sample. Fig. 5 shows the nHA sample’s thermogravimetric analysis (TGA) and differential thermal analysis (DTA) curves. The temperature of the maximum process speed established using the DTA curve analysis (exothermic peaks). Three peaks can be seen in the DTA curve of the nHA sample. The first peak is at 424°C, the second is at 671°C, and the third is 879°C. For temperature up to 424°C, the initial weight loss begins. Water molecules physically adsorbed on the sample could be blamed for this loss.

## DISCUSSION

Living inside the amount of life management and prolongation, synthetic implants of HA and Ca-P ceramics, generally are very common for arduous tissue (e.g., bone) restorations because they accelerate bone growth round the implant. Biological apatites entice interest since it is believed that the various substitutions at the Ca^2+^, PO_4_^3−^ and OH^−^ sites of angular distance and also the presence of many trace components play a critical role within the overall physiological functioning and also the osseointegration method (Rocha *et al*. 2005).

Gómez Vázquez *et al*. (2020) reported that HA was synthesised using echinoderm spines (*Strongylocentrotus purpuratus*) via a precipitation and heat treatment technique at three different temperatures (500°C, 600°C and 700°C). The results showed the impact temperature contend a crucial role in the formation of the HA. As the temperature increased, the scale of the crystals decreased, their resistance decreased and their purity or crystallinity decreased as well (Gómez Vázquez *et al*. 2020). Komalakrishna *et al*. (2017) reported that the nHA from three different marine species was synthesised successfully using the Novel Chemical method. Contrary to our research, the powders, which had a crystalline shape, had a needle-shaped or rod-shaped, and the SEM images also showed that the particle size was found to be in the range of 100 nm to 700 nm (Komalakrishna *et al*. 2017).

HA can be manufactured either in a very artificial manner from analytical grade pure chemicals or natural sources, like corals, sea algae and human/animal bones. However, natural HA is much more preferred than synthetic ones, because most coral-derived apatite’s are resorbable after surgery and promote bone formation much better than semi-restorable HA types. Different marine sources to supply HA/TCP biphasic materials are cuttlefish, numerous nacres, sea urchins, and many others (Agathopoulos *et al*. 2012). This analysis presents a chemical methodology to supply pure, stoichiometry and stable nHA powder using Sea urchin shells and orthophosphoric acid. Seashells usually have aragonite or calcite structure. The nHA powder generated is bulky, since it is formed up of nanocrystalline molecules that form a microcrystalline molecule. Sea urchin shells are composed mainly of calcium carbonate and can be used as the calcium precursor source for HA.

Generally, the shells are heated to 1100°C to remove organic compounds and to convert calcium carbonate to calcium oxide, which upon contact with the atmosphere, is turned to calcium oxide. The calcium hydroxide is then reacted with a valuable phosphate source (such as Diammonium hydrogen phosphate) to produce HA crystals. From the SEM analysis, the prepared HA powder is found to be nanocrystalline. The nanocrystalline HA is often discovered from the images. The produced HA powder has large nature because it is found to be manufactured from nanocrystalline molecules and forms crystalline molecules.

Thermodynamic stability and loss weight of the nHA samples were assessed using thermos gravimetric analysis. Up to 1000°C temperature in the air atmosphere, a heating rate of 10°C/min is used. The thermal analysis study (shown in Fig. 5) shows a weight loss of around 6% up to 400°C temperature, related to absorbed water evaporation, and 6.8% in the range of 400°C to 1000°C. Up to 1000°C, no significant loss was seen. It depicts a change in the recrystallisation state of dehydrated hydroxyapatite at 100°C–900°C. Within the temperature range, more or less stable curvature was found, demonstrating the thermal stability of nHA powder.

## CONCLUSION

In this study, pure nHA has been extracted from sea urchin shells using the hydrothermal method. The used methods are safe and inexpensive. Moreover, the raw materials (sea urchins) feature the advantages of the unlimited source and the biological origin. This is a cost-effective technique for isolating nHA. Most structures are made up of pure calcium carbonate (calcite or aragonite); with the addition of a tiny amount of an organic matrix, HA is synthesised. Onshore and offshore sources of hydroxyapatite are available (such as seaweed, starfish, coral, sea urchin shell, etc.). On Iran’s southern coast, *E. mathaei* is employed as a member of major indigenous sea urchin species. The presence of a carbonated group (which is favourable for biomedical applications) is confirmed by FTIR findings. On the other hand, the functional group research showed that all hydroxyapatite peaks were present, and the product of outstanding purity, according to scanning electron and transmission electron microscopy findings. The powder’s characterisation of this crucial nanomaterial that is valuable in biomedical applications, thermal stability was determined using the TG-DTA method. nHA sea urchin could be a viable biomaterial for biomedical applications in bone tissue engineering. The sea urchin shell is a potential recycling resource for making nHA powder, which can also aid with waste management and environmental protection. Thus, producing nano-sized HA particles from sea urchin shells is possible, and these can be considered good candidates for the new generation of biological implants and drug applications.

## References

[b1-tlsr-34-2-243] Ağaoğullari D, Kel D, Gökçe H, Duman I, Öveçoglu ML, Akarsubaşi AT, Bilgic D, Oktar FN (2012). Bioceramic production from sea urchins. Acta Physica Polonica.

[b2-tlsr-34-2-243] Agathopoulos S, Gunduz O, Sahin YM, Ben-Nissan B, Oktar FN (2012). Nanobioceramics production from razor shell. Key Engineering Materials.

[b3-tlsr-34-2-243] Álvarez-Lloret P, Rodríguez-Navarro AB, Falini G, Fermani S, Ortega-Huertas M (2010). Crystallographic control of the hydrothermal conversion of calcitic sea urchin spine (*Paracentrotus lividus*) into apatite. Crystal Growth & Design.

[b4-tlsr-34-2-243] Cahyaningrum SE, Herdyastuty N, Devina B, Supangat D (2018). Synthesis and characterization of hydroxyapatite powder by wet precipitation method.

[b5-tlsr-34-2-243] Elmi F, Chenarian Nakhaei R, Alinezhad H (2018). Conversion of fisheries waste as magnetic hydroxyapatite bionanocomposite for the removal of heavy metals from groundwater. Journal of Water Science and Technology: Water Supply.

[b6-tlsr-34-2-243] Ettensohn CA (2017). Sea urchins as a model system for studying embryonic development. Reference module in biomedical sciences.

[b7-tlsr-34-2-243] Gómez Vázquez NS, Luque Morales PA, Gomez Gutierrez CM, Nava Olivas OD, Villarreal Sánchez RC, Vilchis Nestor AR, Chinchillas Chinchillas MD (2020). Hydroxyapatite biosynthesis obtained from sea urchin spines (*Strongylocentrotus purpuratus*): Effect of synthesis temperature. Processes.

[b8-tlsr-34-2-243] Hou Y, Shavandi A, Carne A, Bekhit AA, Ng TB, Randy Chi FC, Bekhit AEA (2016). Marine shells: Potential opportunities for extraction of functional and health-promoting materials. Critical Reviews in Environmental Science and Technology.

[b9-tlsr-34-2-243] Khandelwal H, Prakash S (2016). Synthesis and characterization of hydroxyapatite powder by eggshell. Journal of Minerals, Materials Characterization, and Engineering.

[b10-tlsr-34-2-243] Komalakrishna H, Shine Jyoth TG, Kundu B, Mandal S (2017). Low temperature development of nano-hydroxyapatite from *Austromegabalanus psittacus*, star fish and sea urchin. Materials Today: Proceedings.

[b11-tlsr-34-2-243] Mancilla-Sanchez E, Gómez-Gutiérrez CM, Guerra-Rivas G, Soto-Robles CA, Vilchis-Nestor AR, Vargas E, Luque PA (2019). Obtaining hydroxyapatite from the exoskeleton and spines of the purple sea urchin *Strongylocentrotus purpuratus.*. International Journal of Applied Ceramic Technology.

[b12-tlsr-34-2-243] Mohd Pu’ad NAS, Koshy P, Abdullah HZ, Idris MI, Lee TC (2019). Syntheses of hydroxyapatite from natural sources. Heliyon.

[b13-tlsr-34-2-243] Nabavi SM, Shushizadeh MR, Behfar A, Ashrafi MG (2017). Persian Gulf corals: A new hydroxyapatite bioceramics in medicine. International Journal of Pharmaceutical and Phytopharmacological Research (eIJPPR).

[b14-tlsr-34-2-243] Naleway SE, Taylor JRA, Porter MM, Meyers MA, McKittrick J (2016). Structure and mechanical properties of selected protective systems in marine organisms. Journal of Materials Science and Engineering: C.

[b15-tlsr-34-2-243] Ozyegin LS, Sima F, Ristoscu C, Kiyici IA, Mihailescu IN, Meydanoglu O, Agathopoulos S, Oktar FN (2012). Sea snail: An alternative source for nano-bioceramic production. Key Engineering Materials.

[b16-tlsr-34-2-243] Persian Gulf Study Centre (n.d). Geography of Persian.

[b17-tlsr-34-2-243] Rahim SAKA, Nurhasan R (2016). Status of sea urchin resources in the East Coast of Borneo. Journal of Marine Biology.

[b18-tlsr-34-2-243] Rocha JHG, Lemos AF, Agathopoulos S, Valério P, Kannan S, Oktar FN, Ferreira JMF (2005). Scaffolds for bone restoration from cuttlefish. Bone.

[b19-tlsr-34-2-243] Şahin YM, Gündüz O, Bulut B, Özyeʇin LS, Gökçe H, Aʇaoʇullari D, Chou J, Kayali ES, Ben-Nissan B, Oktar FN (2015). Nano-bioceramic synthesis from tropical sea snail shells (tiger cowrie-*Cypraea tigris*) with simple chemical treatment. Acta Physica Polonica.

[b20-tlsr-34-2-243] Shavandi A, Wilton V, Bekhit AEA (2016). Synthesis of macro and micro porous hydroxyapatite (HA) structure from waste kina (*Evechinus chloroticus*) shells. Journal of the Taiwan Institute of Chemical Engineers.

[b21-tlsr-34-2-243] Szatkowski T, Kołodziejczak-Radzimska A, Zdarta J, Szwarc-Rzepka K, Paukszta D, Wysokowski M, Ehrlich H, Jesionowski T (2015). Synthesis and characterization of hydroxyapatite/chitosan composites. Physicochemical Problems of Mineral Processing.

[b22-tlsr-34-2-243] Venkatesan J, Kim SK (2014). Nano-hydroxyapatite composite biomaterials for bone tissue engineering: A review. Journal of Biomedical Nanotechnology.

[b23-tlsr-34-2-243] Venkatesan J, Lowe B, Manivasagan P, Kang KH, Chalisserry EP, Anil S, Kim DG, Kim SK (2015). Isolation and characterization of nano-hydroxyapatite from salmon fish bone. Materials.

